# Efficacy and safety of carbon-ion radiotherapy for lacrimal gland carcinomas with extraorbital extension: a retrospective cohort study

**DOI:** 10.18632/oncotarget.24390

**Published:** 2018-02-03

**Authors:** Kazuhiko Hayashi, Masashi Koto, Hiroaki Ikawa, Kazuhiko Ogawa, Tadashi Kamada

**Affiliations:** ^1^ Hospital of the National Institute of Radiological Sciences, National Institutes for Quantum and Radiological Sciences and Technology, Chiba, Japan; ^2^ Department of Radiation Oncology, Osaka University Graduate School of Medicine, Osaka, Japan

**Keywords:** carbon-ion radiotherapy, lacrimal gland carcinoma, extraorbital extension, safety, efficacy

## Abstract

**Purpose:**

To evaluate the efficacy and safety of carbon-ion radiotherapy (CIRT) for patients with lacrimal gland carcinomas (LGCs) with extraorbital extension

**Results:**

The median follow-up period was 53.7 months. The 5-year local control and overall survival rates were 62% and 65%, respectively. Regarding late toxicities, 12 patients (36.4%) developed Grade 4 optic nerve disorders, including visual losses of the diseased side (*N* = 8; 66.7%), and 1 patient (3.0%) developed a Grade 3 optic nerve disorder. Three patients (9.0%) developed Grade 3 cataracts, 3 (9.0%) developed glaucoma, and 1 (3.0%) developed retinopathy. Two patients (6.1%) had Grade 4 central nervous system necrosis. No Grade 5 late toxicities were observed. The 5-year preservation rate of the ipsilateral eyeball was 86%.

**Conclusion:**

Definitive CIRT is effective for LGCs with extraorbital extension with acceptable toxicity.

**Methods:**

Thirty-three patients treated with CIRT at our institution were analyzed. Sixteen patients (48.5%) had adenoid cystic carcinoma, 8 (24.2%) had adenocarcinoma not otherwise specified, and 9 (27.3%) had other types of the disease. Thirty patients (90.9%) had T4c tumors. The prescribed doses were 57.6 Gy (relative biological effectiveness [RBE]) (*N* = 18; 54.5%) and 64.0 Gy (RBE) (*N* = 15; 45.5%) in 16 fractions.

## INTRODUCTION

Lacrimal gland carcinoma (LGC) is a relatively rare disease with an annual incidence of 0.19 per 1,000,000 population in Denmark [1]. LGC represents a diverse range of histological subtypes, including radioresistant tumors, such as adenoid cystic carcinoma and ductal adenocarcinoma. The most common histological subtype is adenoid cystic carcinoma, which accounts for 50% of all cases of LGC [1]. The therapeutic management of LGCs includes surgery and additional adjuvant chemoradiotherapy or radiotherapy depending on disease stage, histological subtype, the presence or absence of perineural invasion, incomplete surgical resection, involvement of regional lymph nodes, and extracapsular spread [2]. Surgery and adjuvant chemoradiotherapy or radiotherapy has achieved a high 5-year local control rate of > 80.0% [3–5]. However, the majority of the patients with LGCs with extraorbital extension are inoperable. With regard to alternative treatment modalities, photon radiotherapy was shown to be inferior to surgery because histological subtypes, such as adenoid cystic carcinoma and ductal adenocarcinoma, are radioresistant [1, 5], and an effective chemotherapeutic regimen is yet to be established [2]. Hence, there are no effective treatment methods for inoperable LGCs or patients who refuse surgery.

Carbon-ion radiotherapy (CIRT) has a higher linear energy transfer rate than photon radiotherapy and a large relative biological effectiveness (RBE). Therefore, it is considered to be effective for radioresistant tumors. CIRT also has better dose-localizing properties than photon radiotherapy [6]. To date, a dose escalation trial of CIRT in 21 patients with intraorbital LGC, including 16 (76.2%) patients with adenoid cystic carcinoma and 3 (14.3%) with adenocarcinoma, in our institute has shown promising findings [7]. A radiation dose between 48.0 Gy (RBE) and 52.8 Gy (RBE) was delivered in 12 fractions. The 3-year local control and overall survival (OS) rates were 79% and 82%, respectively, although 7 patients (33.0%) lost their vision, and 3 patients (14.3%) developed Grade 3 retinopathies. By contrast, our present study uses 16 fractions, which is generally adopted in CIRT for locally advanced head and neck cancer from a toxicity point of view, because LGCs with extraorbital extension are adjacent to organs at risk, such as the brain and skin [8].

The efficacy and safety of CIRT for LGCs with orbital extension is not well understood. Therefore, this study aimed to evaluate the clinical outcomes of patients who have LGCs with extraorbital extension.

## RESULTS

### Patient, tumor, and treatment characteristics

**Figure 1:**
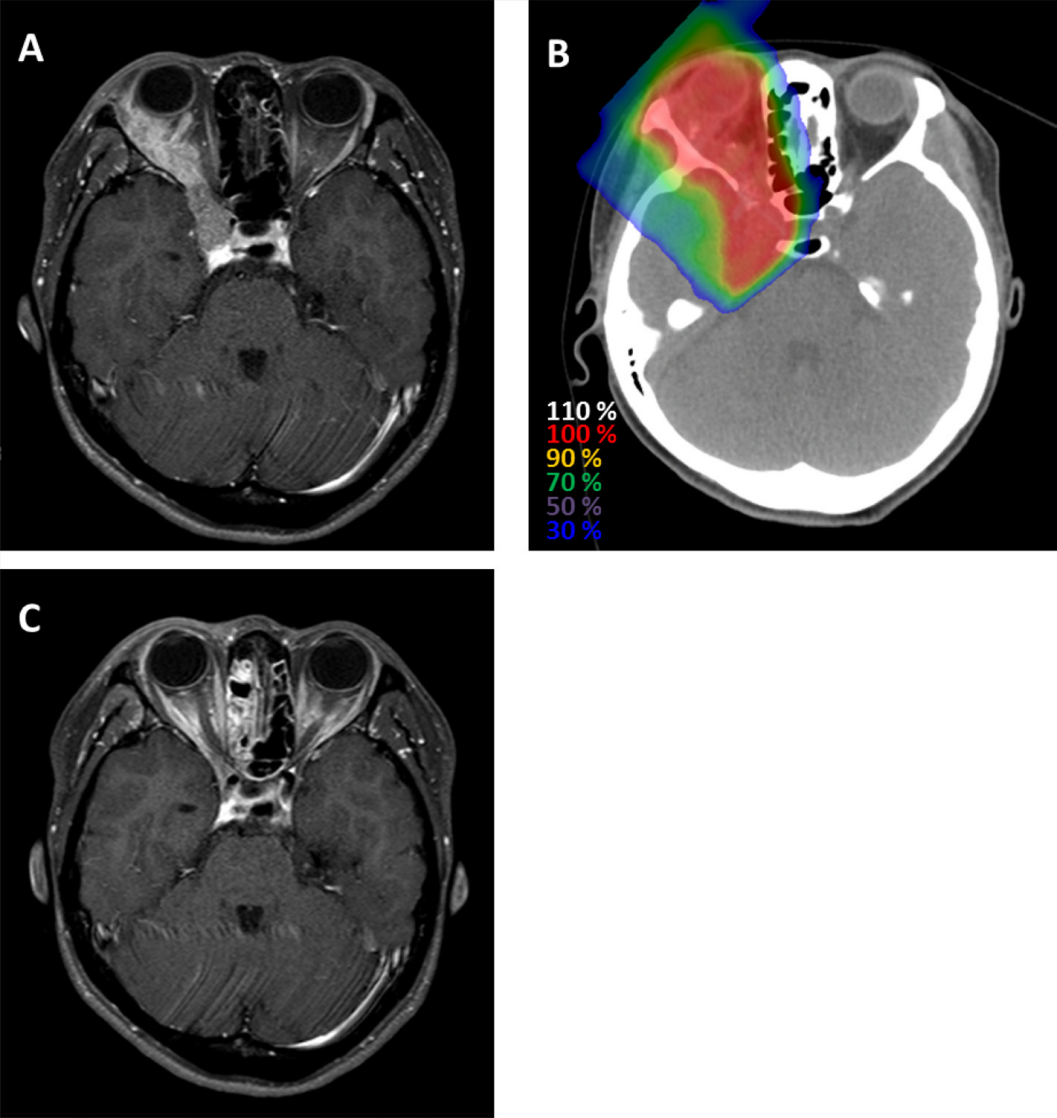
Representative case of a patient with adenoid cystic carcinoma of the right lacrimal gland who was treated with CIRT (**A**) Magnetic resonance imaging before CIRT reveals a well-enhanced right orbital tumor extending from the right lacrimal gland to the right cavernous sinus (**B**) Dose distribution. (**C**) Magnetic resonance imaging 8 months after treatment shows that the tumor had completely disappeared.

The patient, tumor, and treatment characteristics are summarized in Table 1. Sixteen patients (48.5%) had adenoid cystic carcinoma, 8 (24.2%) had adenocarcinoma not otherwise specified, and 9 (27.3%) had other types of the disease. One (3.0%), 2 (6.1%), and 30 (90.9%) patients had T4a tumor, T4b tumors, and T4c tumors, respectively. Thirty-one patients (93.9%) had N0 disease, and 2 patients (6.1%) had N1 disease. Both the 2 patients with N1 disease had parotid lymph node metastases. Their histology was ductal adenocarcinoma (*N* = 1) and sebaceous adenocarcinoma (*N* = 1). None of the patients received concurrent chemotherapy and adjuvant therapy before recurrence and metastasis. Figure 1A–1C shows a representative case of a patient with right LGC who was treated with CIRT.

**Table 1:** Patient and tumor characteristics

Characteristics	Patients (*N* = 33)
Sex, *N* (%)	
M	23 (69.7)
F	10 (30.3)
Age (years), median (range)	58 (23–81)
ECOG PS, *N* (%)	
0	23 (69.7)
1	9 (27.3)
2	1 (3.0)
Histology, *N* (%)	
ACC	16 (48.5)
ADC	8 (24.2)
DA	2 (6.1)
UC	2 (6.1)
SA	2 (6.1)
Other	3 (9.0)
Disease status, *N* (%)	
Initial disease	21 (63.6)
CIRT alone	13 (61.9)
CIRT after surgery	5 (23.8)
CIRT after chemotherapy	3 (14.3)
Recurrent disease	12 (36.4)
Clinical T classification, *N* (%)	
T4a	1 (3.0)
T4b	2 (6.1)
T4c	30 (90.9)
Clinical N classification, *N* (%)	
N0	31 (93.9)
N1	2 (6.1)
Prescribed dose, *N* (%)	
57.6 Gy (RBE) in 16 fx	18 (54.5)
64.0 Gy (RBE) in 16 fx	15 (45.5)
GTV (mL), median (range)	27.9 (1.8–226.4)
CTV (mL), median (range)	92.2 (20.3–322.5)

Abbreviations: ACC, adenoid cystic carcinoma; ADC, adenocarcinoma not otherwise specified; DC, ductal adenocarcinoma; ECOG, Eastern Cooperative Oncology Group; F, female; fx, fraction; M, male; PS, performance status; SA, sebaceous adenocarcinoma; UC, undifferentiated carcinoma.

### Local control and survival

**Figure 2:**
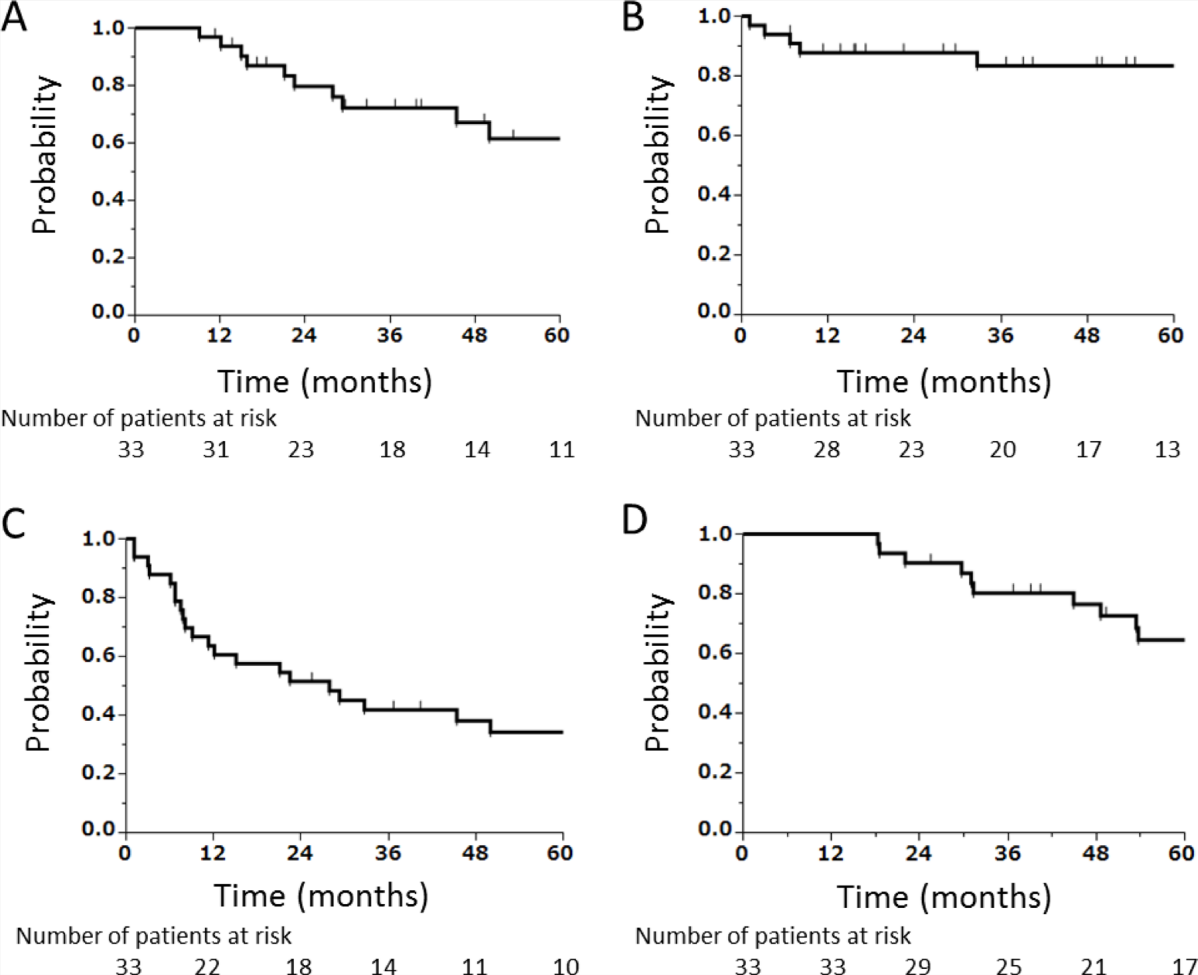
Kaplan–Meier curves (**A**) Local control, (**B**) regional control, (**C**) disease-free survival, and (**D**) OS of all patients combined (*N* = 33).

The median follow-up period of all the 33 patients was 53.7 (range, 13.7–195.4) months. On the last day of observation, 12 patients (36.4%) exhibited local recurrence. Of these patients, 8 developed local recurrence within the planning target volume (PTV), 3 at the boundary of the PTV (cavernous sinus [*N* = 2] and temporal muscle [*N* = 1]), and 1 outside the PTV (within the orbit). Eight patients (24.2%) had regional recurrence, and 15 patients (45.5%) had distant metastases. Thirteen patients (39.4%) had died from their disease, and 4 patients (12.1%) had died from unrelated causes (pneumonitis [*N* = 2], lung cancer [*N* = 1], and Brugada syndrome [*N* = 1]). The 3- and 5-year local control rates were 72% (95% confidence interval [CI]: 53%–86%) and 62% (95% CI: 41%–79%), respectively (Figure 2A), and the 3- and 5-year regional control rates were 83% (95% CI: 65%–93%) and 83% (95% CI: 65%–93%), respectively (Figure 2B). The 3- and 5-year disease-free survival rates were 42% (95% CI: 26%–59%) and 34% (95% CI: 20%–52%), respectively (Figure 2C), and the 3- and 5-year OS rates were 80% (95% CI: 62%–91%) and 65% (95% CI: 45%–80%), respectively (Figure 2D).

Univariate analysis was performed to explore potential prognostic factors for local control and OS among the subgroups (Supplementary Table 1). However, no patient, tumor, or treatment characteristics were found to be prognostic.

### Acute and late toxicities

Regarding acute toxicities, 10 patients (30.3%) developed Grade 2 dermatitis, and 1 patient (3.0%) developed Grade 2 conjunctivitis (Table 2). No acute toxicities of Grade ≥ 3 were observed.

**Table 2:** Toxicities

Toxicities	Grade					Total
	1	2	3	4	5	
Acute toxicities						
Dermatitis	22	10	0	0	0	32
Conjunctivitis	20	1	0	0	0	21
Mucositis	2	0	0	0	0	2
Late toxicities						
Optic nerve disorder	1	0	1	12	0	14
Cataract	5	0	3	0	0	8
Glaucoma	3	1	3	0	0	7
Retinopathy	6	0	1	0	0	7
Vitreous hemorrhage	0	1	0	0	0	1
CNS necrosis	11	0	0	2	0	13
Trismus	1	0	0	0	0	1

Abbreviations: CNS, central nervous system.

Regarding late toxicities, 12 patients (36.4%) developed Grade 4 optic nerve disorders, including visual losses of the diseased side (*N* = 8; 66.7%), and 1 patient (3.0%) developed a Grade 3 optic nerve disorder. The median time after CIRT to the loss of vision in the 8 patients who were affected was 33.5 (range, 10.5–46.3) months. Three patients (9.0%) developed Grade 3 cataracts, 3 (9.0%) developed glaucoma, and 1 (3.0%) developed retinopathy. The patients received cataract surgery, laser therapy, or laser photocoagulation, respectively, that improved the symptoms. None of the patients required an ophthalmectomy owing to ocular or visual toxicities. No ocular or visual toxicities were observed in the healthy side.

Two patients (6.1%) developed Grade 4 central nervous system necrosis, specifically temporal lobe necrosis. One patient (3.0%) developed orientation disturbances due to brain necrosis and edema, which required an emergency necrotomy, and symptoms were improved. The final patient required an Ommaya reservoir to control cystic formation and exacerbation of the brain edema. Afterwards, the reservoir was removed.

Comparison of the clinical target volume (CTV) 1 group and the extended CTV1 group showed that the incidence of Grade 1–3 glaucoma was significantly higher in the extended CTV1 group than in the CTV1 group (40% *vs.* 6%; *P* = 0.03). No significant differences in the incidence of other toxicities were observed between the two groups.

### Eyeball preservation

**Figure 3:**
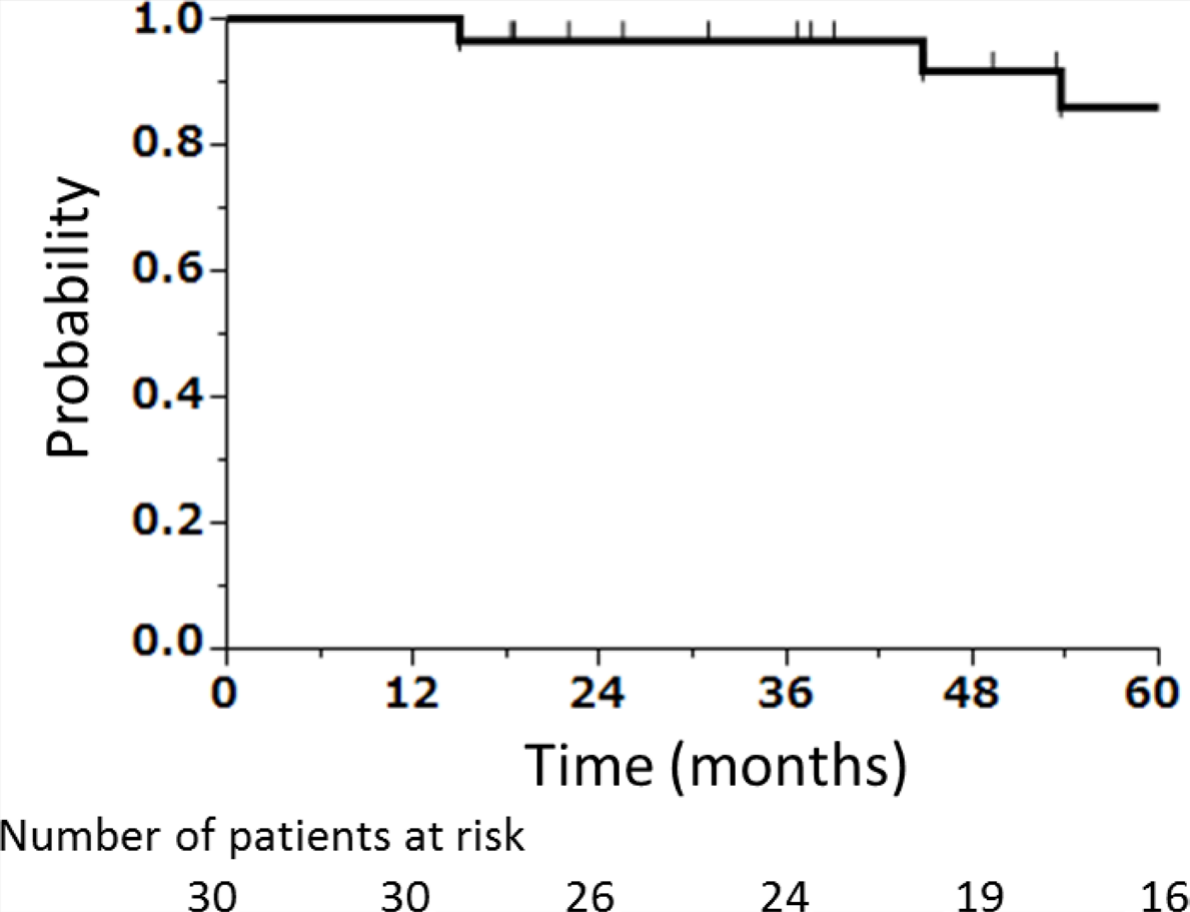
Kaplan–Meier curve of preservation rate of the ipsilateral eyeball after CIRT, excluding patients who had undergone orbital exenteration before CIRT (*N* = 2) and a patient whose treatment information after local recurrence was unclear (*N* = 1)

Two of the 33 patients had already lost the eyeball on their diseased side before CIRT, and one patient missed after local recurrence post CIRT. Of the 30 patients, 10 had local recurrence. Five of these 10 patients were contraindicated for salvage surgery. Of the 10 patients with local recurrences, 7 received salvage local treatment; 2, best supportive care; and 1, chemotherapy. Furthermore, among the 7 patients, 3 underwent orbital exenteration; 2, eye-sparing salvage surgery; and 2, re-CIRT. The 2 patients who received re-CIRT were judged as inoperable. Consequently, 4 patients received eye-sparing salvage surgery or re-CIRT that can preserve the ipsilateral eyes. The 5-year preservation rates of the ipsilateral eyeball were 86% (Figure 3). No operable patients refused salvage surgery.

## DISCUSSION

LGCs that extend into normal tissues are almost unresectable, and the majority of LGCs comprise radioresistant tumors, including adenoid cystic carcinomas and adenocarcinomas not otherwise specified. Therefore, there is no curative treatment modality for LGCs. CIRT for head and neck cancer has already been proven a promising treatment, particularly for patients with radioresistant tumors [9–11]. To the best of our knowledge, this is the first study focusing on LGCs with extraorbital extension. Our findings demonstrate that high local control and an excellent preservation rate of the ipsilateral eyeball are achieved using CIRT. These data suggest that CIRT may be a possible treatment option for LGCs with extraorbital extension.

LGCs are predominantly treated via surgery and adjuvant radiotherapy as necessary. However, surgery is difficult for LGCs with extraorbital extension (e.g., into the intracranial space or temporal muscle). Noh *et al.* [12] treated 19 patients with lacrimal adenoid cystic carcinoma, including 9 with T1–2 disease, 6 with T4b disease, and 4 with T4c disease, using surgery and postoperative radiotherapy. The authors reported a 5-year OS rate of 83%. Tao *et al.* [5] treated 29 patients with T2 (*N* = 3), T3 (*N* = 2), T4a (*N* = 11), T4b (*N* = 1), Tx (*N* = 2), and recurrent (*N* = 10) tumors using orbital exenteration and postoperative photon radiotherapy and reported a 5-year local control and OS rates of 83% and 60%, respectively. The study of Skinner *et al.* [13] included one of the largest cohorts of patients. Forty-six patients who had undergone surgery with or without postoperative photon radiotherapy, including 5 with T1 tumors, 9 with T2 tumors, 25 with T4 tumors, and 7 with Tx tumors, were analyzed. The 5-year locoregional control and OS rates were 60% and 61%, respectively. There have been no reports that have focused on LGCs with extraorbital extension. Therefore, we must compare our findings with those for intraorbital LGCs. Our study revealed that the 3- and 5-year local control and OS rates were 72% and 62% and 80% and 65%, respectively, although 30 patients (90.9%) had T4c tumors. Our data indicate that CIRT may be effective for patients with LGCs who have not received any other curative treatment modality.

Mizoguchi *et al.* [7] reported that for intraorbital tumors, the CTV was defined to within a 5.0-mm region from the boundary of the gross tumor volume (GTV). Accordingly, 3 patients had marginal recurrence. Thereafter, the authors defined the new CTV as the GTV plus the extended margin, including the whole orbit, according to orbital exenteration. Similarly, in our study, CTV1 included the primary lesion with a 5.0–8.0-mm margin, and consequently, 1 patient developed local recurrence inside the orbit, but outside PTV1. Thereafter, we defined the extended CTV1 as CTV1, including the whole orbit. A comparison between the CTV1 and extended CTV1 groups revealed that the incidence of glaucoma was significantly higher in the extended CTV1 group than that in the CTV1 group (*P* < 0.05). However, all patients who had glaucoma improved with conservative treatment. Therefore, the extended CTV1 seemed to be appropriate when we consider the risk of local recurrence.

The incidence of severe late ocular and visual toxicity was not low because the tumor was directly adjacent to organs at risk, such as the ipsilateral optic nerve, lens, and retina, and the whole orbit was included in the high-dose area. Even if our patients had received surgery, most would have undergone orbital exenteration and would have lost the visual function of the diseased side immediately after surgery. Meanwhile, CIRT-induced blindness after CIRT occurred at a median of 33.5 months. Until then, the ipsilateral optical function was preserved. Moreover, visual toxicities of the contralateral side were not observed. Therefore, these visual toxicities seem to be acceptable.

Historically, the standard treatment for LGC has been orbital exenteration in which the eyeball of the diseased side was inevitably removed. More recent evidence is accumulating in support of an eye-preserving local excision, followed by radiotherapy [2, 13]. However, the adaptation is generally considered when tumors of the lacrimal apparatus are smaller and more confined within the orbit [2]. Therefore, for patients with locally advanced LGC with extraorbital extension, who were the focus of this study, orbital exenteration would be appropriate if resection was possible. However, surgical removal of the eyeball and the surrounding tissues substantially changes the patient's physical appearance. Meanwhile, our findings demonstrated a high preservation rate of the ipsilateral eyeball (86% at 5 years), which is largely comparable to that for intraorbital LGCs treated with CIRT (90.4%) [7]. CIRT is effective for maintaining the quality of life of individuals concerned with their physical appearance. When patients with cancer of the lacrimal gland apparatus undergo radiotherapy, the most severe and life-threatening toxicity is central nervous system necrosis. Skinner *et al.* [13] studied surgery and adjuvant photon radiotherapy for cancers of the lacrimal apparatus and reported 3 patients (8%) who developed severe temporal lobe necrosis after radiation and required treatment with surgical intervention. In the present study, although 15 patients (45.5%) had LGCs extending into the intracranial space, only 2 patients (6.1%) experienced Grade 4 central nervous system necrosis due to the improved dose-localization properties of carbon ions compared with photons. Koto *et al.* [14] showed that the brain volume receiving > 50.0 Gy (RBE) was a significant risk factor for the development of Grade ≥2 brain necrosis after receiving CIRT for skull base tumors. Future monitoring of this factor may predict or reduce the incidence of severe central nervous system necrosis.

LGC is histologically similar to salivary gland carcinomas. In a previous study, 46 patients received CIRT for parotid gland carcinomas [15]. Of these patients, 16 (34.8%), 8 (17.4%), 8 (17.4%), and 14 (30.4%) had adenoid cystic carcinomas, adenocarcinomas not otherwise specified, mucoepidermoid carcinomas, and other types of the disease, respectively. Consequently, the 5-year local control rate was 70.1%. Meanwhile, in the present study, 16 (48.5%), 8 (24.2%), and 9 (27.3%) patients had adenoid cystic carcinoma, adenocarcinoma not otherwise specified, and other types of the disease, respectively. The present study showed that the 5-year local control rate was 65% (Supplementary Table 2). The histology and local control rate in the current study are approximately similar to those of previous studies on parotid gland carcinomas.

In this study, 15 patients (45.5%) had distant metastases. Currently, effective systematic chemotherapy has not been established for LGCs. However, Bell *et al*. recently reported that KRAS (Kirsten rat sarcoma) and NRAS (neuroblastoma rat sarcoma) are common in epithelial neoplasms of the lacrimal gland, with the highest rate of mutations found in adenoid cystic carcinoma [16]. Their study indicated that targeted therapy that blocked EGFR (epidermal growth factor receptor)-RAS (rat sarcoma) pathway can be effective. Andreason *et al*. reported that human epidermal growth factor receptor type2 (HER2) and androgen receptor overexpression are common in ductal adenocarcinomas of the lacrimal gland. They suggested that HER2 blockage and androgen deprivation therapy can be a treatment option for such malignancy [17]. In the future, these effective targeted therapeutic modalities are expected to improve the outcomes of LGCs.

This study has two limitations. The first is its retrospective single-institution design, although patients were enrolled prospectively and treated with fixed prescribed doses and fractionation schedules. The second is its limited sample size with only 33 patients included. However, cases of LGC with extraorbital extension are rare, and our cohort size did not differ from those of other studies.

In conclusion, this study demonstrates that definitive CIRT is effective for LGCs with extraorbital extension with acceptable toxicity. However, future large-scale, prospective, multicenter studies are warranted.

## METHODS

### Patients

This study was approved by the Institutional Review Board of our institution. Informed consent has been obtained. A retrospective survey of patients with LGCs with extraorbital extension who had received CIRT at our institution between May 1997 and December 2015 was conducted. Eligible patients were required to have a histologically proven and measurable carcinoma of the lacrimal gland with extraorbital extension [9]. All patients were to be N0M0 or N1M0, with no coexistent malignant active tumor. In total, 33 patients were enrolled. All tumors were reclassified according to the seventh edition of the Union for International Cancer Control tumor-node-metastasis staging system [18].

The National Cancer Institute's Common Terminology Criteria for Adverse Events version 3.0 was the preferred tool for determining toxicities with the highest grade. Acute and late toxicities were defined as those occurring within and later than 3 months of commencing CIRT, respectively.

The histological subtype was classified based on the World Health Organization classification of head and neck tumors (third edition) [19].

### Carbon-ion radiotherapy

Patients were positioned in customized cradles and immobilized using a low-temperature thermoplastic shell. Computed tomography images of all patients fixed in position, using an individually tailored immobilization device, were taken in the supine position. The computed tomography images were used to develop a three-dimensional treatment plan using the in-house HIPLAN software (NIRS, Chiba, Japan) until 2013 and XiO-N (ELEKTA, Stockholm, Sweden and Mitsubishi Electric, Tokyo, Japan) thereafter.

The usual prescribed dose was 64.0 Gy (RBE) delivered in 16 fractions over 4 weeks. When large sections of the skin or brain were included in the target volume, a prescribed dose of 57.6 Gy (RBE) delivered in 16 fractions was selected [9]. Primary lesions were contoured as the GTV on computed tomography images using magnetic resonance imaging as a reference. In the early years of CIRT, the primary lesion plus a 5.0–8.0-mm margin was usually defined as CTV1. Thereafter, CTV1 plus the whole orbit has generally been defined as the extended CTV1. CTV2 is restricted to include the GTV plus a 5.0-mm margin. PTV1, the extended PTV1, and PTV2 are defined as CTV1 and CTV2 plus a 2.0-mm safety margin, respectively, to account for positional uncertainty. In the early years of CIRT, PTV1 was initially irradiated in 9–10 fractions, and PTV2 received a boost to a total dose of 64.0 or 57.6 Gy (RBE) in 16 fractions. Thereafter, the extended PTV1 was initially irradiated in 13/14 fractions, and PTV2 received a boost to a total dose of 64.0 or 57.6 Gy (RBE) in 16 fractions. The dose was prescribed to the isocenter. PTV1 and PTV2 were enclosed conformally at the minimum by the 90.0% isodose line with the prescribed dose. The maximum dose constraint for the stem and healthy side of the optic nerve was 30.0 Gy (RBE). The CTV and PTV margins of areas close to the brain, skin, and anterior portion of the eye were reduced as necessary. Irradiation was performed in 2–5 fields with carbon-ion beams. For each irradiation, the patient's position was confirmed using a computer-aided online positioning system. In N1 cases, metastatic lymph nodes were not treated with CIRT; these were resected after CIRT was performed for the primary lesion.

### Statistical analyses

Local control, regional control, disease-free survival, and OS rates were calculated using the Kaplan–Meier method and compared using the log-rank test. Local control was defined as no evidence of tumor regrowth in the orbit or PTV. Regional control was defined as no evidence of regional lymph node recurrence. Disease-free survival was defined as no evidence of local, regional, or metastatic recurrence. Survival times were calculated from the date of commencing CIRT.

Univariate analysis of the prognostic factors, namely, sex, age, histology, disease status, total dose, and GTV, for local control and OS was performed using the generalized Wilcoxon test. Factors, such as age and the GTV, were used to divide patients into two groups based on the median values. All statistical analyses were conducted using JMP software version 13.0 (SAS Institute Inc., Cary, NC, USA). A two-tailed *P* < 0.05 was considered statistically significant.

Preservation rate of the ipsilateral eyeball was calculated using the Kaplan–Meier method except in 2 patients who previously underwent orbital exenteration before CIRT and in 1 patient whose treatment information after local recurrence was unclear. The preservation rate of the ipsilateral eyeball was defined as the rate of patients with ipsilateral eyeball on the final observation day. The events were defined as removal of the eyeball or local recurrence that was not indicated for salvage surgery or re-irradiation.

Fisher's exact tests were used to compare the incidence of late toxicities between the CTV1 and extended CTV1 groups.

We would also like to thank Editage (www.editage.jp) for English language editing.

## SUPPLEMENTARY MATERIALS TABLES



## Abbreviations

CI: confidence interval
CIRT: carbon-ion radiotherapy
CTV: clinical target volume
GTV: gross tumor volume
LGC: lacrimal gland carcinoma
OS: overall survival
PTV: planning target volume
RBE: relative biological effectiveness.
