# Supplementary material for: Efficacy and safety of carbon-ion radiotherapy for lacrimal gland carcinomas with extraorbital extension: a retrospective cohort study
Source: Oncotarget. 2018 Feb 3;9(16):12932–40. doi: 10.18632/oncotarget.24390 (PMC5849185; doi:10.18632/oncotarget.24390)
Supplement: Supplementary file 1 [file oncotarget-09-12932-s001.pdf]

Efficacy and safety of carbon-ion radiotherapy for lacrimal gland carcinomas with extraorbital extension: a retrospective cohort study

SUPPLEMENTARY MATERIALS

Supplementary Table 1: Univariate analysis for the local control and overall survival rate

Parameters	Patients (*N* = 33)	Local control	Overall survival
		P-value	*P*-value
Sex, *N* (%)		0.648	0.533
M	23 (69.7)		
F	10 (30.3)		
Age (years), *N* (%)		0.155	0.388
≤58	18 (54.5)		
>58	15 (45.5)		
Histology, *N* (%)		0.284	0.739
ACC	16 (48.5)		
ADC	8 (24.2)		
Other	9 (27.3)		
Disease status, *N* (%)		0.165	0.199
Initial disease	13 (39.4)		
Residual tumor	8 (24.2)		
Recurrent disease	12 (36.4)		
Total dose, *N* (%)		0.547	0.195
57.6 Gy (RBE) in 16 fx	18 (54.5)		
64.0 Gy (RBE) in 16 fx	15 (45.5)		
GTV (mL), *N** (%)		0.948	0.745
<27.6	14 (42.4)		
≥27.6	14 (42.4)		

Abbreviations: ACC, adenoid cystic carcinoma; ADC, adenocarcinoma not otherwise specified; F, female; fx, fraction; M, male.
*denotes 28 patients because GTV data of 5 patients were not available.

Supplementary Table 2: Histology and the site of first recurrence

Histology (number)	Local recurrence	Regional recurrence	Distant metastasis		Regional recurrence and distant metastasis		Local recurrence and distant metastasis	
ACC (16)	5	0	3	(Lung, 1; bone, 1; meninges, 1)	1	(Lung metastasis)	1	(Subcutaneous tissue)
ADC (8)	2	2	2	(Bone, 1; dura mater, 1)	0		0	
DA (2)	0	1	1	(Bone and lung)	0		0	
UC (2)	0	1	0		0		0	
SA (2)	1	1	0		0		0	
Other (3)	0	0	1	(Bone)	0		0	

Abbreviations: ACC, adenoid cystic carcinoma; ADC, adenocarcinoma not otherwise specified; DC, ductal adenocarcinoma; SA, sebaceous gland adenocarcinoma; UC, undifferentiated carcinoma.
The parentheses show the site of distant metastasis.
